# 凋亡染色体凝聚诱导因子1在肺癌血小板中的表达及意义

**DOI:** 10.3779/j.issn.1009-3419.2018.09.05

**Published:** 2018-09-20

**Authors:** 琳琳 薛, 丽 谢, 兴国 宋, 现让 宋

**Affiliations:** 250117 济南，山东大学附属山东省肿瘤医院；250002 济南，山东省医学科学院 Shandong Cancer Hospital Affiliated to Shandong University, Jinan 250117, China; Shandong Academy of Medical Sciences, Jinan 250002, China

**Keywords:** 血小板, 肺肿瘤, 剪接体, 凋亡染色体凝聚诱导因子1, mRNA, Platelet, Lung neoplasms, Spliceosome, *ACIN1*, mRNA

## Abstract

**背景与目的:**

在肿瘤发生发展过程中，肿瘤会“教育”血小板引起其mRNAs表达谱改变。由于血小板没有细胞核，其mRNAs的水平变化极可能是通过转录后pre-mRNA的剪接成熟及选择性剪接来实现的。凋亡染色体凝聚诱导因子1（apoptotic chromatin condensation inducer 1, ACIN1）是剪接依赖的多蛋白外显子连接复合物的组成部分之一，参与剪接相关的mRNAs代谢。本研究旨在分析*ACIN1* mRNA在血小板中的表达水平，并探讨其作为肺癌生物标志物的潜力。

**方法:**

收集山东省肿瘤医院156例肺癌患者和58例健康对照作为研究对象。低速离心法获得血小板沉淀，提取总RNA。用RT-PCR技术检测*ACIN1* mRNA在血小板中表达水平，并分析其在肺癌患者和健康对照人群血小板中的表达差异以及*ACIN1* mRNA表达与相关临床因素的相关性。

**结果:**

*ACIN1* mRNA在肺癌患者血小板中的表达水平显著高于健康对照组（*P*=0.015）。受试者工作特征（receiver operating characteristic, ROC）曲线显示*ACIN1* mRNA表达水平检测肺癌的曲线下面积分别是0.608。*ACIN1* mRNA在肺癌血小板中的表达与肺癌患者的年龄、性别、病理类型以及是否转移等无明显关系（*P* > 0.05）。

**结论:**

*ACIN1* mRNA在肺癌患者血小板中显著高表达，其表达水平检测对肺癌的诊断有潜在的临床价值。

## Abstract (en)

**Background and objective:**

During the occurring and developing of tumor, tumor-educated platelets mRNA profiles were altered. Since platelets are anuclear, the level of mRNAs is probably post-transcriptional regulated by the splicing maturation of pre-mRNA and alternative splicing. Apoptotic chromatin condensation inducer 1 (ACIN1) has been shown to be a component of a splicing-dependent multiprotein exon junction complex (EJC) and was involved in mRNA metabolism associated with splicing. This study analyzed the expression of *ACIN1* mRNA in platelets, and explored its potential as a biomarker of lung cancer.

**Methods:**

156 patients with lung cancer and 58 healthy controls in Shandong Cancer Hospital were collected. We isolated platelet pellets by low-speed centrifugation and extracted total RNA. The expression of *ACIN1* mRNA in platelets was detected by RT-PCR, the results were analyzed statistically. And the relationship between expression of *ACIN1* mRNA and clinical factors were also analyzed.

**Results:**

The expression level of *ACIN1* mRNA in platelets of patients with lung cancer was significantly higher than that in platelets of healthy controls (*P*=0.015). The ROC curve showed that the area under the curve of ACIN1 mRNA for detecting lung cancer were 0.608. The expression of *ACIN1* mRNA in platelets of lung cancer has no significant relationship with age, gender, pathological type and metastasis or not (*P* > 0.05).

**Conclusion:**

*ACIN1* mRNA was highly expressed in platelets of lung cancer patients, and the detection of its expression level might have potential clinical value for the diagnosis of lung cancer.

肺癌是全世界范围内发病率和死亡率最高的恶性肿瘤之一，其起病隐匿，早期临床表现缺乏特异性，大部分患者在确诊时已处于局部晚期或发生远处转移，预后往往较差^[1, 2]^。

血小板是起源于骨髓巨核细胞的多功能无核细胞碎片，在外周血中其含量仅次于红细胞。在肿瘤患者血液循环中血小板包裹循环肿瘤细胞（circulating tumor cells, CTCs）保护其不受血流剪切力的损伤，并协助CTCs形成转移。这些受肿瘤“教育”的血小板（tumor-educated platelets, TEPs）^[3, 4]^的功能发生了明显改变，表明其基因的表达有所改变。Calverley等^[5]^使用微阵列分析了7名健康个体和5例未治疗转移性肺癌患者的血小板mRNAs表达谱，发现了200个mRNAs的表达水平有显著的改变。因为血小板没有细胞核，其mRNAs的水平变化不可能是在转录水平被调控的，最大的可能是通过转录后pre-mRNA的剪接成熟及选择性剪接来实现的^[6, 7]^。凋亡染色体凝聚诱导因子1（apoptotic chromatin condensation inducer 1, ACIN1）是剪接依赖的多蛋白外显子连接复合物的一个组成部分，直接参与剪接相关的mRNAs代谢，TEPs中众多mRNAs表达水平的改变预示着参与mRNAs剪接代谢的蛋白的表达有可能发生改变。

本研究通过比较肺癌患者及健康对照血小板*ACIN1* mRNA的表达水平，以验证以上推论，并对其作为肺癌生物标志物的潜力进行初步探讨。

## 1 材料与方法

### 1.1 一般资料

山东省肿瘤医院自2016年12月-2017年4月经组织活检病理证实的首诊肺癌患者156例，中位年龄62.5（范围30-89）岁，男性111例，女性45例。非小细胞肺癌119例，其中腺癌78例，鳞癌41例，临床分期Ⅰ期11例，Ⅱ期3例，Ⅲ期40例，Ⅳ期65例。小细胞肺癌37例，其中局限期15例，广泛期22例。同期体检健康个体58例为对照组，中位年龄58.5（范围40-73）岁，男性27例，女性31例。

### 1.2 总RNA提取及其浓度和质量检测

在实验前应准备好焦炭酸二乙酯处理水处理好的Ep管、枪头等器材，避免RNA酶污染。按照Invitrogen^TM^ TRIzol^TM^ Reagent操作指导提取总RNA，最终用20 μL无核酶水溶解RNA。用分光光度仪（ASP-3700）分析RNA浓度和纯度，RNA琼脂糖凝胶电泳测RNA的完整性。

### 1.3 荧光定量RT-PCR检测*ACIN1* mRNA的表达

第一步逆转录，将PrimeScript^TM^ RT reagent Kit with gDNA Eraser（TaKaRa）试剂盒内试剂解冻混匀，配置20 μL反应体系，保持37 ℃ 15 min，85 ℃ 5 s合成cDNA。第二步PCR扩增，*ACIN1*上游引物5’-AGGTTAGGCAAGGAGGTGGT-3’，下游引物5’-TGTTCCCAAG AGAAGGCTGT-3’；内参*ACTB*上游引物5’-TTAGTTGCGTTACACCCTTTC-3’，下游引物5’-GCTGTCACCTTCACCGTTC-3’，由生工生物工程（上海）股份有限公司设计合成。PCR扩增在Roche LightCycler®480上进行，每个样品均做复孔，PCR扩增体系：10 μL FastStart PCR Master（ROCHE）、1 μL Super Green荧光染料（北京金博益生物技术有限公司）、0.5 μL上游引物、0.5 μL下游引物、2 μL cDNA溶液、6 μL无核酶水，反应条件：95 ℃预变性5 min，95 ℃变性5 sec、60 ℃复性30 s、72 ℃延伸20 s共45个循环，97 ℃ 5 s、65 ℃ 1 min、95 ℃ 15 s检测溶解曲线。绘制荧光扩增曲线和溶解曲线，得出Ct值，应用ΔCt法（ΔCt=目的RNA Ct值-内参RNA Ct值）分析荧光定量PCR结果，ΔCt值表示目的RNA的相对表达量，ΔCt值越小表明目的RNA表达越强。

### 1.4 统计学处理应用SPSS

22.0统计软件进行数据分析，计量资料比较若服从正态分布用*t*检验并以平均数±标准差表示；若不服从正态分布用非参数检验并以中位数，四分位间距表示。*P* < 0.05为差异有统计学意义。绘制受试者工作特征（receiver operating characteristic, ROC）曲线，并计算ROC曲线下面积（area under the curve, AUC）以评估指标的诊断价值。

## 2 结果

### 2.1 血小板纯度检测

血小板RNA含量远少于白细胞，需尽可能减少白细胞的数量以免对血小板RNA谱造成污染。在光学显微镜下用牛鲍计数板计数总血小板和白细胞的数量证实低速离心法获得的富血小板血浆纯度为每10^6^血小板有0个-5个白细胞，与文献报道一致^[8]^。对全血自然沉降2 h和低速离心法获得血浆涂片进行瑞氏染色在高倍镜下观察，发现自然沉降获得血浆在多个高倍镜视野下均有多少不等的白细胞出现，而低速离心法获得的富血小板血浆在多个高倍镜视野下均无白细胞出现。见图 1。

**1:**
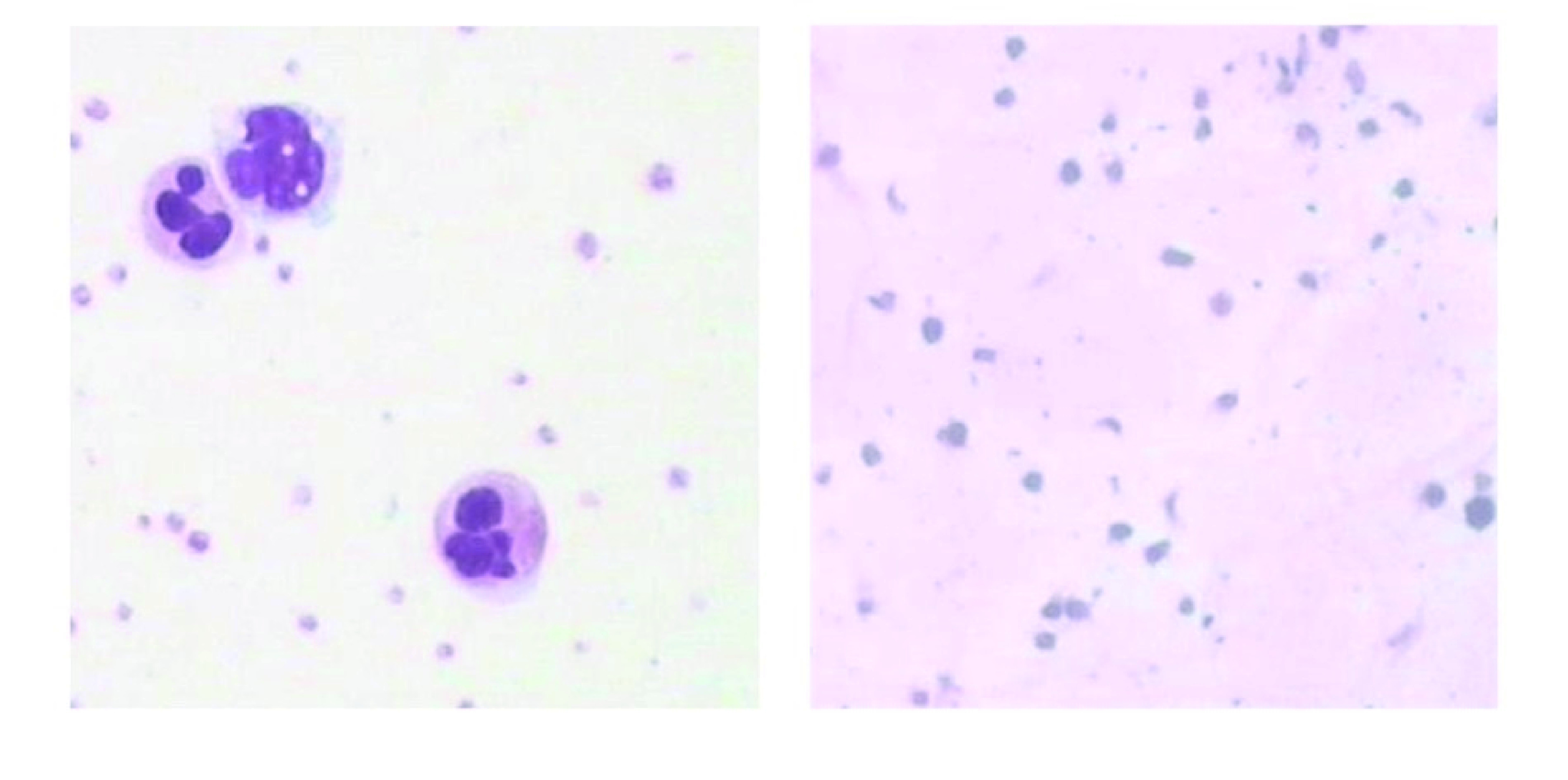
自然沉降时血浆高倍镜下（瑞氏染色，100×）观察到血小板和白细胞（左），低速离心时血浆高倍镜下（瑞氏染色，100×）观察到血小板富集而无白细胞（右）。 Platelets and leukocytes were observed in plasma which was obtained by natural sedimentation (left, Wright's stain, 100×); enriched-platelets without leukocytes were observed in plasma which was obtained by low-speed centrifugation (right, Wright's stain, 100×).

### 2.2 RNA浓度和质量检测

样品用分光光度计测定D260和D280，D260:D280值均在1.8-2.0之间，此时测定的RNA浓度 > 20 ng/μL。取10 μL总RNA溶液进行电泳鉴定总RNA完整性，在紫外线下可见18 s及28 s两条略明亮的条带，未见5 s处有明亮清晰的条带。

### 2.3 *ACIN1* mRNA在肺癌患者与健康对照血小板中表达水平的比较

肿瘤“教育”血小板RNA谱发生了明显改变，可能是通过转录后pre-mRNA的剪接成熟及选择性剪接调控实现的。ACIN1是剪接体的一个组成部分，直接参与剪接相关的mRNAs代谢，其表达水平有可能发生改变。因此我们检测了156例肺癌患者和58例健康对照血小板中*ACIN1* mRNA的表达水平。我们对214例血小板样本提取总RNA后使用逆转录试剂盒合成cDNA，并进行RT-PCR反应，获得*ACIN1*及内参*ACTB*的Ct值，并对其求ΔCt值。*ACIN1* mRNA在肺癌患者血小板中的表达水平显著高于健康对照（*P*=0.015）。*ACIN1* mRNA在非小细胞肺癌患者血小板中表达水平显著高于健康对照（*P*=0.022），在小细胞肺癌患者血小板中的表达水平高于健康对照但差异无统计学意义（*P*=0.058），见图 2。

**2:**
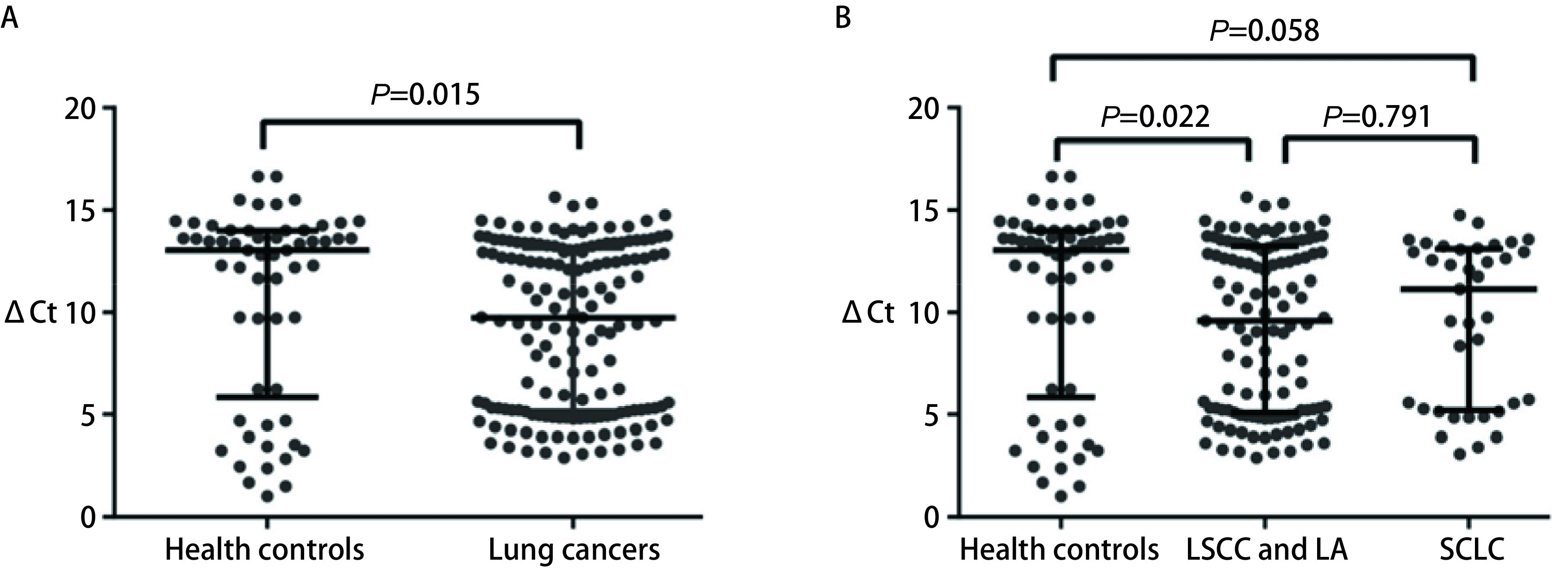
ACIN1 mRNA在血小板中的表达水平。A：*ACIN1* mRNA表达水平在肺癌患者和健康对照血小板中的比较；B：*ACIN1* mRNA表达水平在鳞癌和腺癌患者、小细胞肺癌患者及健康对照血小板中的比较。ΔCt：*ACIN1* mRNA的相对表达量；Health controls：健康对照；LSCC：肺鳞癌；LA：肺腺癌；SCLC：小细胞肺癌。 The expression levels of *ACIN1* mRNA in platelets. A: The comparison of *ACIN1* mRNA expression levels in platelets between lung cancers and healthy controls; B: The comparison of *ACIN1* mRNA expression levels in platelets among squamous cell carcinoma and adenocarcinoma patients, small cell lung cancers patients, and healthy controls. ΔCt: relative expressions of *ACIN1* mRNA; LSCC: lung squamous cell carcinoma; LA: lung adenocarcinoma; SCLC: small cell lung cancer.

### 2.4 *ACIN1* mRNA表达水平检测肺癌的效能

*ACIN1* mRNA表达水平检测肺癌的ROC曲线AUC为0.608，尤登指数最大为0.275，此时Δct为13.438，即ΔCt < 13.438时为肺癌。血小板*ACIN1* mRNA表达水平检测129例/156例为肺癌，敏感性为0.827；26例/58例为健康，特异性为0.448，总的准确性为0.724。见表 1。

**1:** *ACIN1* mRNA表达水平检测肺癌与病理组织检测肺癌一致性比较 Agreement comparison in detecting lung cancer by testing *ACIN1* mRNA levels and tumor tissues

		Tumor tissues		Total
		Positive	Negative	
The levels of *ACIN1* mRNA	ΔCt < 13.438	129	32	161
	ΔCt≥13.438	27	26	53
Total		156	58	91

### 2.5 *ACIN1* mRNA在肺癌血小板中的表达与临床相关因素分析

*ACIN1* mRNA在肺癌血小板中的表达与年龄、性别、病理类型以及是否转移等无关（*P* > 0.05），见表 2。

**2:** *ACIN1* mRNA的表达与临床相关因素的关系 Relationships between expression of *ACIN1* mRNA and clinical factors

Clinical factors	*n*	Positive [*n*(%)]	*P*
Gender			
Male	111	88 (79.30)	
Female	45	41 (91.11)	0.351
Pathological type			
Adenocarcinoma	78	64 (82.05)	
Squamous cell carcinoma	41	32 (78.05)	
Dmall cell carcinoma	37	33 (89.19)	0.956
Age (yr)			
> 60	85	70 (82.35)	
≤60	71	59 (83.10)	0.347
Metastasis			
No	69	60 (86.96)	
Yes	87	69 (79.31)	0.259

## 3 讨论

肺癌是临床常见的恶性肿瘤之一，5年生存率为17.4%，转移性肺癌患者因错过了最佳治疗时机，5年生存率只有4.2%^[9]^。目前，癌胚抗原、糖类抗原125和细胞角蛋白19片段是肺癌检测和诊断常用的分子生物标志物，但这些生物标志物在晚期肺癌诊断中比早期肺癌更准确^[10]^。多种基于血液的癌症相关生物资源已用于癌症诊断的研究中，其中基于血液检测的CancerSEEK评估多种癌症中循环蛋白的表达水平和cfDNA的突变用于癌症的早期诊断^[11]^，还包括cfRNA、代谢产物、胞外囊泡和循环肿瘤细胞，血小板也成为其中的一员。

已有研究^[12]^表明，血小板是肿瘤细胞生长、侵袭、扩散、迁移、渗出和建立远处转移的关键参与者。血小板RNA对外界刺激做出快速反应的动态特性使得以TEPs RNA表达谱为基础的液体活检用于癌症诊断成可能。2015年Best等^[3]^对55例健康个体和228例不同肿瘤类型的癌症患者（包括非小细胞肺癌、结直肠癌、胰腺癌、成胶质细胞瘤、乳腺癌和肝胆癌）血小板样本进行RNA测序的研究表明TEP RNA表达谱发生改变并可用于癌症诊断，准确率为96%。2017年Best等^[7]^应用TEP RNA表达谱和群体智能算法鉴定非小细胞肺癌患者和非癌志愿者，准确率可达81%。

肿瘤患者外周血中TEPs可能处于“半激活”状态^[7]^，血小板高反应性，可启动血小板内信号传导，导致血小板pre-mRNA的剪接成熟以及选择性剪接事件的发生。研究报道选择性剪接过程在癌症中处于紊乱状态，一些剪接因子作为癌基因直接促进癌症的发生和进展，其表达可能上调或下调^[13]^。剪接因子SR（serine and arginine rich）蛋白家族是RNA生成的重要调控因子，参与RNA的组成型表达、选择性剪接、mRNA的转运和翻译，其中富含丝氨酸和精氨酸的剪接因子6（serine and arginine rich splicing factor 6, SRSF6）在结肠癌和肺癌中过度表达^[14]^。*ACIN1*编码的蛋白通过与AAC-11的相互作用干扰抗癌药物诱导的细胞死亡^[15]^，它还是mTORC1潜在的作用靶点^[16]^，在血小板中是剪接体的组成部分，参与mRNA剪接相关的代谢，是肿瘤患者血小板被“教育”的执行者之一，其表达水平可能会发生改变。本研究分析了146例肺癌患者和58例健康对照*ACIN1* mRNA在血小板中的表达水平，发现*ACIN1* mRNA在肺癌患者血小板中表达水平显著高于健康对照。此外，*ACIN1* mRNA表达水平检测肺癌的敏感性为0.827，特异性为0.448，准确性为0.724。因此，推论血小板*ACIN1* mRNA具有作为新的特异性分子生物标志物用于肺癌诊断的潜能。

血小板RNA表达谱是动态变化的，并与癌症的发展和转移密切相关，具有成为特异性肿瘤生物标志物的潜能，可能为检测肿瘤、个体化治疗以及评估预后提供指导。
